# Physician Interactions Associated With Increased Reception of Substance Use Disorder Treatment

**DOI:** 10.7759/cureus.76950

**Published:** 2025-01-05

**Authors:** Abigail M Smurzynski, Jeffrey R Gardere, Olalekan Ogunsakin

**Affiliations:** 1 Behavioral Health, Touro College of Osteopathic Medicine, New York, USA; 2 Pathology, Touro College of Osteopathic Medicine, New York, USA

**Keywords:** alcohol, interactions, physician, recommendations, substance use disorder, treatment

## Abstract

Objectives

According to the 2020 National Survey on Drug Use and Health (NSDUH), only 10% of the estimated 40 million individuals with substance use disorder (SUD) sought treatment. In this analysis, we aimed to examine the factors and characteristics of individuals who had received SUD treatment to gain a better understanding of why some patients attend treatment while the majority do not.

Methods

We performed a retrospective analysis of publicly available data from the 2020 NSDUH, accessed through the Substance Abuse and Mental Health Service Administration (SAMHSA) using IBM SPSS Statistics for Windows, Version 27.0 (Released 2020; IBM Corp., Armonk, New York, United States). We limited our analysis to individuals who self-reported having an alcohol or illicit drug use disorder in the preceding year. We examined the relationship between physician interactions and reception of SUD treatment at any facility in the preceding year (2019-2020) or in their lifetime using the chi-squared analysis and odds ratios (OR) with a 95% confidence interval (CI) and 0.05 alpha level.

Results

Traditional physician inquiries regarding the amount and frequency of substance use were not significantly associated with treatment receipt (p>0.05). However, more personalized interactions were significantly associated with treatment receipt (p<0.05). For example, individuals who reported an interaction where a physician asked if they had any drinking problems had a 2.52-fold increase in the odds of treatment receipt in 2020 (OR: 2.52; 95% CI: 3.7-1.7; p<0.001), and individuals who reported an interaction where a doctor advised them to cut down on drinking were 3.86 times more likely to receive treatment in 2020 (OR: 3.86; 95% CI: 5.85-2.55; p<0.001). Moreover, individuals who reported an interaction with a doctor where they were offered information on alcohol treatment had a fourfold increase in treatment receipt in 2020 (OR: 4.84; 95% CI: 7.54-3.1; p<0.001) and a threefold increase over a lifetime (OR: 3.20; 95% CI: 4.62-2.22; p<0.001); however, only 9.6% of individuals reported such an interaction.

Conclusions

Personalized and informative discussions offering information on SUD treatment options and cessation strategies were associated with higher rates of treatment reception compared to traditional inquiries about the amount and frequency of substance use.

## Introduction

Substance use disorder (SUD) affects approximately 48.5 million people aged 12 or older (or 17.1% of the population) in the United States including 28.9 million people with alcohol use disorder, yet only 2.3 million people or 7.9% received alcohol use disorder treatment in the past year [1,2]. The impact of this lack of treatment is responsible for an estimated 178,307 annual deaths and 4,126,082 alcohol-related emergency department visits in the United States in 2023 [1,2]. The financial impact of the cost of alcohol use disorder to the United States was estimated to be $249 billion in 2010 [3]. 

This gap in SUD care has been prevalent for many years: the 2017 Center for Behavioral Health Statistics and Quality (CBHSQ) Short Report stated that "despite the benefits of treatment, research suggests that few Americans receive any or adequate substance use treatment" [4]. The same report demonstrated that from 2002 to 2014, the percentage of adults aged 18 years or older who received SUD treatment at a specialty facility, which is defined as a drug and alcohol rehabilitation facility (inpatient or outpatient), hospital (inpatient only), or mental health center [5], remained relatively stable at around 7.6% of adults with a past year SUD [4]. Similarly, in 2015, an estimated 2.3 million people aged 12 or older received treatment at a specialty facility in that past year, representing 10.8% of the 21.7 million people who were identified as having an SUD in 2015 [6]. Additionally, fewer than one in 10 adults who identified as having a need for alcohol use disorder treatment reported that they received treatment services from 2015 to 2018 [7]. In 2019, of the 21.6 million people aged 12 or older who qualified for SUD treatment, only 12.5% (or 2.7 million people) received some form of SUD treatment [8,9]. While some may be quick to point to the COVID-19 pandemic as a major source of the current gap in general medical and SUD treatment, it is clear from these prior investigations that this issue was prevalent long before the COVID-19 pandemic [4,6,8,9].

Gaps in the treatment of SUD, including in the areas of patient communication and physician education, are often influenced by a combination of factors, including medical, psychological, social, and financial determinants [10-13]. Previous investigations have revealed significant disparities in the reception of substance abuse treatment comparing race and ethnicity, age, and sex [14,15]. When controlling for individual characteristics, the odds of Black and Hispanic patients completing SUD treatment were significantly lower than their White counterparts [14-16]. To a similar effect, previous studies have revealed that men were consistently 6-10% more likely to complete SUD treatment from 2006 to 2017 compared to women [7,15,16]. In addition, a treating physician expressing to a patient a need for SUD treatment alone has not been associated with the receipt of treatment in past studies [9]. In this analysis, we aimed to examine the factors and characteristics of individuals who had received SUD treatment in the past year or at any point in their lifetime and gain a better understanding of why certain patients receive treatment while the majority do not.

This article was previously presented as a poster at the 2024 Annual Addiction Health Services Research Conference on October 16, 2024, and at Touro University Research Day on April 4, 2024. 

## Materials and methods

Data were retrospectively analyzed from the National Survey on Drug Use and Health (NSDUH) conducted from January to March 2020 and July through December 2020 (data collection was interrupted due to the COVID-19 pandemic) [17-19]. The NSDUH 2020 data set was accessed through the Substance Abuse and Mental Health Service Administration (SAMHSA) and analyzed using IBM SPSS Statistics for Windows, Version 27.0 (Released 2020; IBM Corp., Armonk, New York, United States) [19]. This national survey was conducted through in-person and web-based interviews of persons 12 years and older over a one-year period starting in 2020 [17-19]. Among the 32,893 individuals included in the original NSDUH survey sample, 1557 were utilized for our data analysis [17-19]. First, we chose 18 variables of interest from the NSDUH data set for analysis. Then, respondents who indicated "yes", that is, they suffered from an alcohol or illicit drug use disorder in the past year (2019-2020), were included (n=5343). Respondents who answered "never used alcohol" or "did not use alcohol in the past 12 months" were excluded (n=345). Finally, the missing values which were encoded by the NSDUH and were encoded in the data set when downloaded were excluded from analysis (n=3441). After all inclusion and exclusion criteria outlined above were met, the total data set used for analysis contained 1,557 individuals. This was determined to be Non-Human Subject Based Research (NHSR) by the TouroNY IRB (protocol number: 22478).

Primary analyses examined frequencies and cross-tabulation between reception of SUD treatment with race, sex, age, income level, and interactions with healthcare professionals. SUD treatment was defined by the NSDUH and self-reported by individuals as having received treatment for their use of alcohol or illicit drugs at any location such as a hospital (inpatient), a rehabilitation facility (inpatient or outpatient), a mental health center, an emergency room, a private doctor's office, a self-help group, prison/jail, or some other place [19]. Bivariate and multivariate logistical regression models to further explore these associations with treatment receipt were initially attempted, but due to multicollinearity, we were unable to complete those analyses. A stepwise logistic regression was performed (backward selection, p>0.05 for removal) which revealed physician interactions as the most significant predictor of treatment receipt. We focused only on physician interactions in the subsequent analysis due to the strength of association and elimination of other factors due to multicollinearity.

In the secondary analysis, cross-tabs were performed with the chi-squared analysis, and odds ratios (OR) were calculated to analyze the unique predictive contribution of clinical interactions to treatment reception. OR and associated 95% confidence intervals (CI) are reported at a 0.05 alpha level. Post hoc testing was performed using the Bonferroni correction method, and the p-values reported have been adjusted and reflect this correction and multiplication factor.

## Results

Primary analysis

Among the 32,893 individuals included in the original NSDUH survey sample, 1557 were utilized for analysis [17-19]. Table 1 examined the race, age, and income on treatment reception among individuals identified with alcohol or illicit drug use disorder. Individuals who received treatment in the past year (2019-2020) and at any point in their lifetime were more likely to be non-Hispanic White (n=80 (69.6%) and n=195 (75%), respectively), male (n=71 (61.7%) and n=162 (62.3%), respectively), and above the age of 26 (n=84 (73.1%) and n=184 (70.8%), respectively) compared to those who did not receive treatment (p<0.05). There was no significant difference in the income levels between those who received treatment and those who did not (p>0.05). Multivariate stepwise logistic regression revealed clinician-based interactions to be the most significant predictor of treatment reception.

**Table 1:** Demographic characteristics of persons with alcohol or illicit drug use disorder in 2019-2020

Sample characteristics and group comparisons (n=1557)	Overall, N (%)	Received treatment at any location for illicit drug or alcohol use in 2019-2020, N (%)		Received treatment for illicit drug or alcohol use in their lifetime, N (%)	
		No	Yes	No	Yes
Race					
Non-Hispanic White	1119 (71.9)	1039 (72.1)	80 (69.6)	924 (71.2)	195 (75)
Non-Hispanic Black	108 (6.9)	103 (7.1)	5 (4.3)	98 (7.6)	10 (3.8)
Non-Hispanic Other	113 (7.3)	102 ( 7.1)	11 (9.6)	91 (7)	22 (8.5)
Non-Hispanic Asian	30 (1.9)	30 (2.1)	0 (0)	27 (2.1)	3 (1.2)
Hispanic	187 (12)	168 (11.7)	19 (16.5)	157 (12.1)	30 (11.5)
Total	1557 (100)	1442 (100)	115 (100)	1297 (100)	260 (100)
Age category					
12-17 years	71 (4.6)	63 (4.4)	8 (7)	61 (4.7)	10 (3.8)
18-25 years	703 (45.2)	680 (47.2)	23 (20.0)	637 (49.1)	66 (25.4)
26-34 years	375 (24.1)	334 (23.2)	41 (35.7)	296 (22.8)	79 (30.4)
35 years or older	408 (26.2)	365 (25.3)	43 (37.4)	303 (23.4)	105 (40.4)
Total	1557 (100)	1442 (100)	115 (100)	1297 (100)	260 (100)
Total family income					
Less than $20,000	325 (20.9)	293 (20.3)	32 (27.8)	264 (20.4)	61 (23.5)
$20,000-49,999	428 (27.5)	395 (27.4)	33 (28.7)	343 (26.4)	85 (32.7)
$50,000-74,999	230 (14.8)	214 (14.8)	16 (13.9)	189 (14.6)	41 (15.8)
$75,000 or more	574 (36.9)	540 (37.4)	34 (29.6)	501 (38.6)	73 (28.1)
Total	1557 (100)	1442 (100)	115 (100)	1297 (100)	260 (100)
Gender					
Male	692 (44.4)	621 (43.1)	71 (61.7)	530 (40.9)	162 (62.3)
Female	865 (55.6)	821 (56.9)	44 (38.3)	767 (59.1)	98 (37.7)
Total	1557 (100)	1442 (100)	115 (100)	1297 (100)	260 (100)

Secondary analysis

Among the 1557 individuals who reported alcohol use disorder or illicit drug use disorder from 2019 to 2020, 115 (7.4%) reported that they received treatment at any facility/location from 2019 to 2020. Table 2 describes the association between treatment reception in 2019-2020 and physician interactions. Table 3 describes the association between treatment reception at any point in an individual's lifetime and physician interactions. Among the 1557 total individuals, 260 (16.7%) reported reception of treatment in their lifetime.

**Table 2:** Clinical interactions associated with reception of treatment at any location for alcohol or illicit drug use from 2019 to 2020 among those with alcohol or illicit drug use disorder The chi-squared analysis and Bonferroni correction method were used to generate the p-values OR: odds ratio; CI: confidence interval

Clinical interactions	Did not receive treatment/unknown (n=1442), N (%)	Received treatment in 2019-2020 (n=115), N (%)	OR	Upper 95% Cl	Lower 95% Cl	P-value
The doctor asked how often you drink	1201 (83.3)	89 (77.4)	0.687	1.09	0.434	1.71
The doctor asked how much you drink	1172 (81.3)	91 (79.1)	0.874	1.40	0.547	9.10
The doctor asked if you have any drinking problems	375 (26)	54 (47)	2.52	3.70	1.71	<0.001
The doctor advised you to cut down on drinking	175 (12.1)	40 (34.8)	3.86	5.85	2.55	<0.001
The doctor offered info about alcohol treatment	115 (8)	34 (29.6)	4.84	7.54	3.10	<0.001
The health professional discussed my drug use with me	584 (40.5)	60 (52.2)	1.6	2.35	1.1	0.229
The health professional asked about the past 12 months of drug use	1227 (85.1)	102 (88.7)	1.37	2.49	0.758	4.70
The health professional asked about the past 12 months of alcohol use	1336 (96.8)	105 (91.3)	0.346	0.705	0.17	0.037

**Table 3:** Clinical interactions associated with reception of treatment at any location for alcohol or illicit drug use in their lifetime for persons with alcohol or illicit drug use disorder from 2019 to 2020 The chi-squared analysis and Bonferroni correction method were used to generate the p-values OR: odds ratio; CI: confidence interval

Clinical interactions	Did not receive treatment/unknown (n=1297), N (%)	Received treatment in their lifetime (n=260), N (%)	OR	Upper 95% Cl	Lower 95% Cl	P-value
The doctor asked how often you drink	1078 (83.1)	212 (81.5)	0.897	1.27	0.635	8.56
The doctor asked how much you drink	1055 (81.3)	208 (80)	0.918	1.28	0.657	9.87
The doctor asked if you have any drinking problems	322 (24.8)	107 (41.2)	2.12	2.79	1.6	<0.001
The doctor advised you to cut down on drinking	144 (11.1)	71 (27.3)	3.01	4.16	2.18	<0.001
The doctor offered info about alcohol treatment	96 (7.4)	53 (20.4)	3.20	4.62	2.22	<0.001
The health professional discussed my drug use with me	529 (40.8)	115 (44.2)	1.15	1.51	0.880	4.85
The health professional asked about the past 12 months of drug use	1102 (85)	227 (87.3)	1.22	1.81	0.820	5.23
The health professional asked about the past 12 months of alcohol use	1257 (96.9)	244 (93.8)	0.485	0.881	0.268	0.242

Secondary analysis showed that individuals who reported interactions where a doctor offered information on alcohol treatment had 4.84 higher odds of receiving treatment in the past year (2019-2020) and 3.02 times higher odds of receiving treatment in their lifetime compared to those who had no such interactions. Likewise, individuals who had interactions where a doctor advised them to cut down on drinking were 3.86 times more likely to receive treatment in the past year (2019-2020) and 3.01 times more likely to receive treatment in their lifetime than those who did not report such interactions. Additionally, individuals who had an interaction where a doctor asked if they had any drinking problems had 2.52 times higher odds of receiving treatment in the past year (2019-2020) and 2.12 times higher odds of receiving treatment in their lifetime compared to those with no such interaction. The most frequent interaction reported was a doctor asking how much you drink (n=1290 (82.9%)), while the least frequent interaction reported was a doctor offering information on treatment (n=149 (9.6%)). See Table 4. Interactions where a doctor asked how much or how often you drink had no significant association with the reception of treatment in the past year (2019-2020) or reception of treatment in their lifetime (p>0.05) (see Table 2 and Table 3). 

**Table 4:** Frequency of clinical interactions among persons with alcohol or illicit drug use disorder from 2019 to 2020

Clinical interactions	Yes (n=1557), N (%)	No (n=1557), N (%)
The doctor asked how often you drink	1290 (82.9)	267 (17.1)
The doctor asked how much you drink	1263 (81.1)	294 (18.9)
The doctor asked if you have any drinking problems	429 (27.6)	1128 (72.4)
The doctor advised you to cut down on drinking	215 (13.8)	1342 (86.2)
The doctor offered info about alcohol treatment	149 (9.6)	1408 (90.4)
The health professional discussed my drug use with me	644 (41.4)	913 (58.6)
The health professional asked about the past 12 months of drug use	1329 (85.4)	228 (14.6)
The health professional asked about the past 12 months of alcohol use	1501 (96.4)	56 (3.6)

## Discussion

In this study, we found that those who received treatment in the past year (2019-2020) or at any point in their lifetime were more likely to be non-Hispanic White, male, and above the age of 26. This is consistent with previous studies [14,15]. Surprisingly, there was no significant difference in the income levels between those who received treatment and those who did not. This suggests that other factors may have more influence on individuals' treatment decisions. Additionally, a gender disparity was observed with the majority of those seeking treatment being male, consistent with prior investigations [7-10]. It is important to note that the only genders available for survey participants to report were male and female. While other gender options exist, they were not included in the source data, and we acknowledge this limitation. Further examination into the impact of income and gender could be explored in future studies.

Our secondary analysis demonstrated a significant association between reception of treatment and interactions where doctors asked about any drinking problems, advised individuals to cut down on drinking, and offered information about alcohol treatment (p<0.05). Additionally, no significant correlation was found between questions that asked about the frequency and quantity of alcohol consumed and receipt of SUD treatment. These findings suggest that while current recommendations emphasize screening tools, for example, the US Preventative Services Task Force (USPSTF) recommends screening all adults 18 years or older for "unhealthy alcohol use" using the Alcohol Use Disorders Identification Test-Consumption (AUDIT-C) screening instrument, there are opportunities to guide clinicians on how to engage therapeutically with patients after identifying an SUD [20].

Moreover, a notable discrepancy was observed in how consistently screening providers address these key aspects of alcohol use during patient interactions. Specifically, individuals with SUD were asked about alcohol amount and drinking frequency about three to eight times more often than they were asked about drinking problems, advised to cut down on drinking, or offered information on treatment options (Table 4). Furthermore, the most significant association was observed between the reception of treatment and interactions where a doctor offered information about alcohol treatment, and yet, offering information about treatment was reported at the lowest frequency among all the interactions analyzed (Table 4). This variability in the frequency of healthcare provider interactions may reflect physician time constraints, patient dishonesty about substance use, and/or variability in physician SUD education and comfort levels.

One contributing factor may be the limited time physicians have with their patients [21]. As a result, many providers are pressed for time and may skip discussions about alcohol cessation and information about treatment options, unaware that providing information on alcohol treatment could increase the likelihood of treatment receipt by three to four times (see Table 2 and Table 3). Another possible explanation for the variability in physician interactions is patient dishonesty about substance use, which could result in false negative screenings leading to fewer discussions on cessation and treatment. However, the significant increase in the likelihood of receiving treatment when providers ask about drinking problems suggests that patient dishonesty may not fully explain these inconsistencies (see Table 2 and Table 3).

Another plausible explanation is a variability in physician training regarding SUD. For instance, a national survey conducted in 2000 to determine the amount of formal training in SUD that occurs in graduate medical education (GME) programs found that the percentage of programs with required SUD training ranged from 95% in psychiatry to 31.8% in pediatrics, with 56.3% for all programs combined [22]. The wide assortment of programs and inconsistent teachings could result in varying degrees of knowledge regarding SUD. A needs assessment at the University of Chicago conducted during the 2018-2019 academic year revealed that 86% of Internal Medicine residents felt their instruction during residency on SUD was "none or too little" and they did not feel prepared to treat SUD [23]. The lack of consistent SUD education in physician training creates the opportunity for patients with SUD to go without further interventions after screening is performed.

This analysis is not without its limitations. The 2020 NSDUH data used in this analysis does not include information on the nature of interactions, such as the location (e.g., emergency department vs. primary care), the demeanor of the individuals (e.g., attitude, disposition, and behaviors), or the length of time spent in the physician's office, factors that could potentially influence why an interaction may occur in one scenario versus another. While the data specifies when interactions occur between doctors as opposed to other healthcare professionals, it shows that interactions where healthcare professionals asked about the past 12 months of alcohol use were associated with a decreased odds of receiving treatment. However, these findings may have been skewed by the small sample size of individuals who did not report such interactions, potentially driving the significance toward a negative association. Additionally, given that this data was collected during 2020, it is important to consider the potential impact of COVID-19 on healthcare delivery. While most of the survey questions inquired about "the past year" meaning 2019 into 2020, and most surveys were completed before March 2020, provider concerns about COVID-19 exposure and contact time may have impacted the frequency of healthcare provider interactions in this analysis [17-19]. Additional research is necessary to better understand the impact of physician interactions on SUD treatment reception. This includes exploring the influence of patient-physician relationships, the amount of time physicians can dedicate to patient care, and the role of SUD education level.

While we train doctors and other healthcare professionals to ask about the quantity and frequency of alcohol consumption, we must also ensure they are comfortable taking the next steps when a patient is suspected of or clearly has a substance abuse problem. One potential solution is to reinvigorate provider education and continuing education, emphasizing the importance of patient interactions, alcohol and drug cessation, and various treatment options for SUD, including motivational interviewing, medications, outpatient or inpatient treatment, specific harm reduction strategies, and 12-step programs [24,25]. Training programs in which health professional students learned about the culture and efficacy of 12-step recovery meetings by sitting in on them, as well as being taught behavioral change counseling methods, were associated with more positive attitudes toward persons with SUD and interprofessionalism [26-28]. In addition, a scoping review aimed to investigate peer-reviewed literature on SUD education in medical schools globally found that 91% of the studies identified positive associations between educational interventions relating to SUDs with an improvement in medical students' skills, knowledge, and attitudes [29]. Through improved education efforts, we can make physicians more comfortable in addressing SUD during the doctor-patient interaction and collectively make a positive difference for patients with SUD.

In addition, physicians, psychologists, and other healthcare professionals should be taught the importance of SUD treatment as part of holistic medical care and highlight its impact on the body as a unit. Through the increased treatment of SUD, we can improve health outcomes in multiple fields as substance addiction is associated with one or more health issues, which could include lung or heart disease, stroke, cancer, and mental health conditions [30]. 

## Conclusions

In this analysis, individuals who received SUD treatment in the preceding year (2019-2020) and/or at any point in their lifetime were more likely to have been offered information on alcohol treatment, given alcohol cessation advice, and/or asked if they had a drinking problem by a physician. Interestingly, no significant association was identified between interactions where an individual was asked how much or how often they consume alcohol and the reception of treatment. Yet, these interactions occurred three to eight times more often than those directly correlated with treatment receipt. This finding illustrates that despite the regular performance of screenings for alcohol and illicit drug use disorder by physicians, follow-up questions, such as those we investigated, that are significantly correlated with treatment receipt are not routinely carried out (Table 4). However, this analysis did not provide the contextual factors present during these patient-physician interactions, and thus, further investigation is needed to better contextualize the impact of physician interactions, patient-physician relationships, motivational interviewing, and physician education level on patient motivation and reception of SUD treatment.
